# Application the mass spectrometry MALDI-TOF technique for detection of *Babesia canis canis* infection in dogs

**DOI:** 10.1007/s00436-014-4124-1

**Published:** 2014-09-20

**Authors:** Łukasz Adaszek, Tomasz Banach, Michał Bartnicki, Dagmara Winiarczyk, Paweł Łyp, Stanisław Winiarczyk

**Affiliations:** 1 Department of Epizootiology and Infectious Diseases, Faculty of Veterinary Medicine, University of Life Sciences Lublin, 30 Głęboka St., 20-612 Lublin, Poland; 2 Department and Clinic of Animal Internal Diseases, Faculty of Veterinary Medicine, University of Life Sciences, 30 Gleboka St., 20-612 Lublin, Poland

**Keywords:** *Babesia canis canis*, Diagnostic, Mass spectrometry, Tick-borne diseases

## Abstract

The aim of this study was to use rapid mass spectrometry (MS)-based proteomics analyses for diagnosis of *Babesia canis canis* infections in dogs. The study was conducted on two groups of dogs—healthy dogs and dogs infected with *B. canis canis* which demonstrated symptoms of babesiosis. The matrix-assisted laser desorption ionization time-of-flight (MALDI-TOF) MS technique revealed the presence of a protein fraction of 51–52 kDa in the blood serum of all the animals infected with the protozoa, which was not found in the serum of healthy dogs. The proteins are suspected to be disease markers, whereas the MALDI-TOF technique itself has high specificity and sensitivity and can be applied in analytical laboratories in the diagnosis of canine babesiosis.

## Introduction

Canine babesiosis is a common and clinically significant tick-borne disease caused by hematozoan parasites of the genus *Babesia* (Homer et al. 2000). Standard diagnosis of babesiosis is the identification of *Babesia* parasites in Giemsa-stained thin-film blood smears examined by microscopy. However, the detection of *Babesia* parasites is difficult in dogs with unapparent or chronic infections since the level of parasitemia is very low (Müller et al. 2010). The PCR technique and its varieties (real-time PCR, LAMP, etc.) are characterised by relatively high sensitivity in identifying protozoa invasions. However, these methods may also present false-negative results of blood tests, if the parasites accumulate in the spleen, for instance (Adaszek and Winiarczyk 2010; Adaszek et al. 2013). Therefore, the development of a highly specific and sensitive system for the diagnosis of *Babesia canis* infection is required.

Mass spectrometry (MS)-based proteomics analyses offer exciting new approaches to identify biomarkers for the detection of disease and for monitoring therapeutic and toxic outcomes. Matrix-assisted laser desorption ionization time-of-flight (MALDI)-based proteome profiling of serum, other biofluids, and tissue sections has been widely and aggressively employed for pattern-based diagnostics and biomarker discovery. MALDI spectral features correspond to a subset of proteins present in the sample and collectively constitute proteomic patterns that represent different biological states (Meiser et al. 2010; Ndao 2012; Ahmad et al. 2012).

The aim of the study was to apply the MALDI time-of-flight (MALDI-TOF) technique to demonstrate changes in the serum protein profile of dogs infected with *B. canis*.

## Materials and methods

### Animals used in the study

The study included 22 mixed breed dogs (9 females and 13 males), aged 1–9 years, divided into two groups. Group 1, the control group (*n* = 7; 4 males and 3 females), consisted of healthy dogs. Group 2, the study group (*n* = 15; 5 females and 10 males), consisted of dogs naturally infected with *B. canis canis*. All dogs in the latter group showed symptoms of babesiosis (apathy, anorexia, changes of urine colour, paleness of mucous) and tested positive in thin blood smears and in PCR for *B. canis canis* parasite.

Full blood samples were collected from animals in both groups into test tubes containing a coagulation accelerator, which were then centrifuged to obtain serum that was used for proteomic testing.

### MALDI-TOF MS

Serum samples of 50 μl were vortexed, diluted tenfold, and then cleansed on 0.2-μl Zip-Tip microcolumns (Merck Chemicals) according to a standard procedure (TN 226) that included preliminary activation of the stationary phase with H_2_O:acetonitrile (ACN) solutions (Merck Chemicals). The prepared serums were mixed with the sinapinic acid (SA) matrix suspended in a TA 30 solution 30 (70:30 0.1 % TFA in H_2_O:ACN). A layer of the SA matrix suspended in EtOH HPLC Grade (Merck Chemicals) was placed on a MTP Polished Steel holder (Bruker). After the matrix dried, the test samples were placed on analytical spots (50:50 sample:SA in TA 30). Three analyses with the ultrafleXtreme mass spectrometer (Bruker) were performed for each sample within the weight range of 20 to 100 kDa. The spectrometric analysis was conducted using the flex Control 3.3 (build 108) programme, while the spectra were analysed with the flex Analysis 3.3 (build 80) programme.

## Results and discussion

**Fig. 1:**
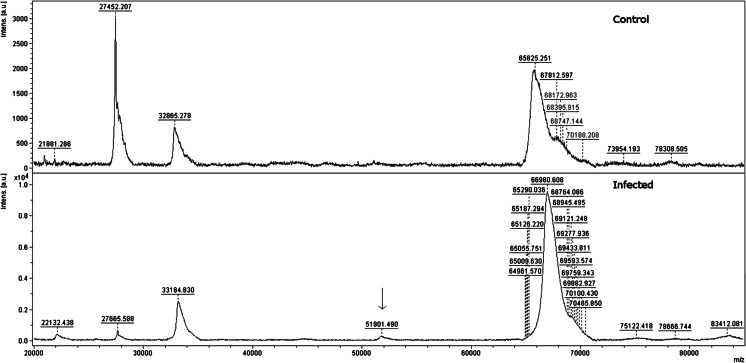
MALDI-TOF mass spectrometry analysis of blood serum from a healthy and infected by *Babesia canis canis* parasite patient. Spectra were acquired in linear positive mode in a mass range of 20–100 kDa. An additional protein fraction of approximately 51–52 kDa (*arrow*) was visible in serum of dogs with babesiosis

Proteomic analysis demonstrated the presence of eight protein fractions ranging from 20 to 100 kDa in serum samples obtained from all the tested animals, both in the study group and in the control group. Moreover, an additional protein fraction of approximately 51–52 kDa was found in all the serum samples obtained from the dogs with babesiosis (Fig. 1). These proteins were not found in the serum of any of the control group dogs. This indicates that the fraction discussed may be used to differentiate between healthy dogs and animals infected with *B. canis* protozoa and it may be considered an infection marker. The analysis of mass spectograms of tested serum samples and the comparison of the control group and the test group spectra did not reveal any other significant differences (Fig. 1).

There is only one literature report available regarding the use of mass spectrometry in testing protein profiles of *B. canis*-infected dog serum. The results obtained by the authors indicated that the technique may be used to monitor the clinical course of babesiosis and to study the pathogenesis of the disease (Kuleš et al. 2014). However, there is no data available regarding the use of the MALDI-TOF mass spectrometry for the diagnostics of *B. canis* infections in dogs. Own observations indicate that MALDI-TOF MS is a sensitive and a reliable diagnostic technique. Achieved test results were 100 % compliant with molecular (PCR) testing. It is worth emphasizing that the costs of the test are much lower, and time required to prepare a sample is shorter than in the case of standard molecular testing. Own calculations demonstrate that the cost of testing one serum sample collected from a dog with suspected babesiosis is approximately 5 euro and the time needed for the assay does not exceed 2 h.

The protein fraction of a similar weight—51–52 kDa—as has been observed in own studies on the serum of *B. canis*-infected dogs, was found in SPA antigen obtained from the supernatant of the protozoa in vitro culture (Adaszek et al. 2012a). The fraction appeared to be strongly immunogenic, which was confirmed in Western blot tests. It was the proteins of 51–52 kDa that caused the strongest reaction with serum samples obtained from the dogs vaccinated with SPA (Adaszek et al. 2012a, b). Considering the above, it may be inferred that these proteins are released into a dog’s serum by the protozoa after infection and they result in a signal in the infected dogs’ serum, which may then be treated as a disease marker. However, to achieve complete certainty as to the origin of the 51- to 52-kDa fraction, it is recommended to conduct further analysis of its composition according to the adopted top-down analytical strategy.
